# Characterization of the Molecular Mechanisms of Resistance against DMI Fungicides in *Cercospora beticola* Populations from the Czech Republic

**DOI:** 10.3390/jof7121062

**Published:** 2021-12-11

**Authors:** Ram Kumar, Jana Mazakova, Asad Ali, Vishma Pratap Sur, Madhab Kumar Sen, Melvin D. Bolton, Marie Manasova, Pavel Rysanek, Miloslav Zouhar

**Affiliations:** 1 Department of Plant Protection, Faculty of Agrobiology, Food and Natural Resources, Czech University of Life Sciences Prague, Kamycka 129, 165 00 Prague, Czech Republic; kumar@af.czu.cz (R.K.); mazakova@af.czu.cz (J.M.); aliasad@af.czu.cz (A.A.); manasova@af.czu.cz (M.M.); rysanek@af.czu.cz (P.R.); 2 Laboratory of Reproductive Biology, Institute of Biotechnology of the Czech Academy of Sciences, BIOCEV, Prumyslova 595, 252 50 Vestec, Czech Republic; VishmaPratap.Sur@ibt.cas.cz; 3 Department of Agroecology and Crop Production, Faculty of Agrobiology, Food and Natural Resources, Czech University of Life Sciences Prague, Kamycka 129, 165 00 Prague, Czech Republic; senm@af.czu.cz; 4 Northern Crop Science Laboratory, United States Department of Agriculture, 1307 18th St N, Fargo, ND 58102, USA; melvin.bolton@usda.gov

**Keywords:** *Cercospora beticola*, DMI fungicide resistance, *Cyp51*, molecular dynamics simulations, real-time PCR

## Abstract

Cercospora leaf spot (CLS), caused by the fungal pathogen *Cercospora beticola*, is the most important foliar pathogen of sugar beet worldwide. Extensive reliance on fungicides to manage CLS has resulted in the evolution of fungicide resistance in *C. beticola* worldwide, including populations in the Czech Republic. One important class of fungicides used to manage CLS is the sterol demethylation inhibitors (DMI). The aim of our study was to assess DMI resistance in *C. beticola* from the Czech Republic and elucidate the molecular basis of DMI resistance in this population. A total of 50 isolates were collected in 2018 and 2019 from the major sugar beet growing regions of the Czech Republic and assessed for in vitro sensitivity to the DMI fungicides propiconazole, prochloraz, and epoxiconazole. These analyses identified three strains that exhibited 50% effective concentration (EC_50_) values > 1.0 μg mL^–1^ against respective fungicides, which were therefore considered resistant. In contrast, strains that exhibited lowest EC_50_ values were considered sensitive. To explore the molecular basis of resistance in these three strains, the cytochrome P450-dependent sterol 14α-demethylase (*Cyp51*) gene was sequenced. Sequence analysis identified a Y464S mutation in all three resistant strains. To assess whether *Cyp51* gene expression may play a role in DMI resistance, selected strains were grown in vitro with and without fungicide treatment. These analyses indicated that *Cyp51* gene expression was significantly induced after fungicide treatment. Thus, we conclude that Y464S point mutation along with induced *Cyp51* gene overexpression is likely responsible for resistance against DMI fungicides in *C. beticola* from the Czech Republic.

## 1. Introduction

Cercospora leaf spot (CLS), caused by *Cercospora beticola*, is one of the most damaging foliar diseases in *Beta vulgaris L.* (sugar beet) [1,2]. If left unmanaged, this disease can cause losses of up to 50% of the yield and sugar content [3,4,5]. Symptoms of CLS are necrotic leaf spots of around 2–3 mm that eventually coalesce in the later stages of the disease development resulting in large necrotic regions on the leaf surface, thus reducing the photosynthetic activity of affected leaves, which effects the yield [2]. While host resistance is an important means of managing CLS, fungicides are critical tools to manage this disease in most sugar beet growing regions of the worldwide [6,7]. 

In the last decades, a loss of sensitivity against sterol demethylation inhibitors (DMI), quinone outside inhibitors (QoIs), and methyl benzimidazole carbamate (MBC) fungicides for management of *C. beticola* has been reported [3,8,9,10,11,12]. DMIs are categorized as medium-risk fungicides by the Fungicide Resistance Action Committee (FRAC). Reduced sensitivity of *C. beticola* towards DMI has occurred in some populations [11,13,14]. Hence, researchers across the globe must monitor *C. beticola* population for fungicide resistance.

DMI fungicides are an important group used to manage CLS worldwide [2]. This fungicide targets the cytochrome P450-dependent sterol 14α-demethylase (*Cyp51*) enzyme, which catalyses the demethylation of lanosterol to produce an important precursor of ergosterol that is a vital component of fungal cell membranes [15]. Consequently, inhibition of *Cyp51* gene by DMI fungicides results in fungal cell death [16]. A single point mutation in *Cyp51* gene at L144F/I309T/Y464S has been associated with a loss of sensitivity against DMI in *C. beticola* [8]. These resistance mechanisms exhibit qualitative resistances; point mutation leads to reduced sensitivity towards particular fungicide [17,18].

Fungicide resistance can be grouped into two basic mechanisms: target-site and non-target-site based resistance. Target-site-based resistance mainly involves single or multiple nucleotide polymorphisms, increased target gene amplification, or target gene overexpression [19,20,21]. Nontarget site-based resistance reduces the concentration of active fungicide that interacts with the target site protein, primarily by reducing translocation and vacuolar sequestration, enhancing metabolism and/or reducing absorption [22]. The active elimination of the fungicide by over-expressed of ATP-binding cassette (ABC) transporters was reported to be responsible for a loss of sensitivity towards DMI in field populations of *Penicillium digitatum, Botrytis cinerea,* or *Zymoseptoria tritici* [23,24,25]. The target protein might be affected by the fungicide, but the active elimination of the fungicide separates the fungicide from the target protein [18,26,27]. Moreover, more than one mechanism might confer resistance within a species, within a population, and even within a single individual [14].

Currently the exact mechanism of resistance is unknown for the Czech Republic isolates. Hence, the objective of this study was to characterize DMI resistance in *C. beticola* strains collected from the Czech Republic. In this study, we had screened 50 isolates collected from sugar beet fields across the Czech Republic. Thereafter, the *Cyp51* gene sequence of strains displaying the highest and the lowest EC_50_ values were analysed. Three resistant strains with reduced sensitivity towards propiconazole, prochloraz, and epoxiconazole were identified. Two strains were detected to be sensitive against all three DMI fungicides tested. In addition, we had examined over-expression of the target *Cyp51* gene as complementary resistance mechanism. We had investigated fungicide induced and constitutive over-expression of the *Cyp51* gene via RT-qPCR. Additionally, molecular docking and dynamics simulation studies were also performed to gain insights into associated mechanism underlying the resistance. The results obtained from our study will provide a basis for future research using comparative reverse genetics, transcriptomics and epigenetic approaches.

## 2. Materials and Methods

### 2.1. Sample Collection

Leaves with CLS symptoms were collected from different fields across the Czech Republic. To maintain heterogeneity among the samples, we used different collection bags to collect and store the samples from individual fields. Samples were processed in the laboratory of the Department of Plant Protection, Czech University of Life Sciences Prague, immediately after collection, on the same day. Visible leaf spots from each leaf sample were cut with a cork borer (20 mm). After cutting, the individual leaf pieces were disinfected with 20% bleach, and the leaves were further washed three times (two minutes each) in three different beakers with distilled water. Once the leaf section was washed, it was transferred in the glass petri plates on which we used two layers of wet filter paper (with distilled water) and kept for 72 h at 24 °C and 12 h of light. Moreover, we had checked moisture content in each petri plates after 48 h to ensure that there as enough moisture. After 72 h, we picked single hyphal tip transfer (using microscope in the laminar flow hood) and transferred it to the potato dextrose agar (PDA; HiMedia, Mumbai, India) [28]. After two weeks, the petri plates were stored at 4 °C for future experiments.

### 2.2. Fungicide Sensitivity Assay

Overall, 50 strains were screened against three different DMI fungicides, propiconazole, prochloraz, and epoxiconazole. The active ingredient of each fungicide was obtained from Sigma-Aldrich, St. Louis, MO, USA. The fungicides were dissolved in dimethyl sulfoxide (DMSO; St. Louis, MO, USA) to prepare the stock solution. Fungicide dose-response assays were conducted in PDA petri plates with three replicates and serial 10-fold dilutions from 0.001 μg/mL to 10 μg/mL. A total of three strains (R2, R5, and R10) had EC_50_ value more than 10 μg/mL for propiconazole, prochloraz, and epoxiconazole, respectively (Supplementary Table S1). These strains were re-examined using serial 10-fold dilution from 0.001 to 100 μg/mL to measure their exact EC_50_ value. The experiment was repeated twice for each concentration. After 14 days, inhibition of radial growth was measured and compared with that of the untreated control according to Wong and Wilcox [29]. EC_50_ value were calculated for all three fungicides using GraphPad Prism software (9.0.0) for Windows OS (GraphPad Software, San Diego, CA, USA), based on the mean colony diameter and radial growth of each strain. The resistance factor (RF) was calculated as the ratio of the EC_50_ of each R strains and the S strains. The isolate/s with EC_50_ > 1.0 ug/mL were considered as resistant [30]. Based on this, we had screened the collected *C. beticola* isolates from the Czech Republic. Thereafter, based on the highest EC_50_ value and visual observation of mycelial growth reduction, we had opted to continue the study with the highest EC_50_ as resistant (R) and EC_50_ ≤ 0.5 ug/mL values for all three DMI fungicide considered as sensitive (S) isolates.

### 2.3. Preparation of Liquid Cultures

Liquid cultures of *C. beticola* were used for over-expression experiments. For this study, 50 mL potato dextrose broth (PDB; HiMedia, Mumbai, India) was inoculated with a piece of mycelium scraped in a 100mL flask, taken from the leading edge of a PDA media culture plate. The culture was incubated for 72 h at room temperature on shaker at 200 rpm. For fungicide treatment 50 μL of each DMI fungicide were added into their respective strain stock solution {10 mg/mL in dimethyl sulfoxide (DMSO; St. Louis, MO, USA)} for a final concentration of 10 μg/mL in four biological replicates. As non-treated controls each strain was amended with 50 μL DMSO (Sigma-Aldrich, St. Louis, MO, USA) without any fungicide. The flasks were shaken for an additional two days at 200 rpm, and the mycelium was harvested using funnel and cheesecloth [29].

### 2.4. C. beticola Cyp51 Gene Expression Analysis

RNA from fresh mycelial samples were obtained from the liquid culture. RNA was isolated using a Hybrid-R^TM^ kit (GeneAll Biotechnology Co., Ltd., Seoul, Korea). A High-Capacity cDNA Reverse Transcription Kit (Applied Biosystems™, Waltham, MA, USA) was used to reverse-transcribe the RNA templates. *Cyp51* gene expression experiments were conducted in a CFX Connect Real-Time PCR Detection System (Bio-Rad Laboratories, Hercules, CA, USA). cDNA (~13 ng) was used as templates for expression analysis, respectively. *Actin* was used as an internal standard [29]. Gene-specific primers (GSPs) for quantitative real-time PCR experiments are listed in Table 1. The results were calculated using the 2^−ΔΔCt^ method [31,32], and a comparison between the S and R strains was performed using a two-sample t-test. The RT-qPCR experiment was repeated twice and in four replications.

### 2.5. Cloning of the Whole Cyp51 Gene from C. beticola

Mycelial samples from each R and S strain were collected for total genomic DNA (gDNA) extraction. The GenElute™ Plant Genomic DNA Miniprep Kit (Sigma-Aldrich, St. Louis, MO, USA), following the manufacturer’s instructions, was used for the extraction of gDNA from the fungal strains. Prior to the extraction, the mycelial samples were scraped from PDA plates and transferred to 250 mL flasks containing 50 mL of PDB. The setup was kept on a shaker (200 rpm) at 24–26 °C for 96 h. After 96 h, the mycelial samples were filtered through cheesecloth (using a funnel) and used for gDNA extraction. *Cyp51* gene-specific primers were designed based on the publicly available sequence of the *Cyp51* gene from *C. beticola* (GenBank Acc. No. KU665583.1). The list of primers is described in Table 1. PCR was performed using a C1000 thermocycler (Bio-Rad, Hercules, CA, USA) with 25 ng of total gDNA per reaction. The thermocycler was programmed at an initial denaturation step at 95 °C for 5 min, followed by 40 cycles of 5 s at 95 °C, 10 s at 58 °C to 61 °C (based on the annealing temperature of the primer pair), and 2 min at 72 °C along with a final extension step for 10 min at 72 °C. The PCR-amplified products were separated in a 1.5% agarose gel and subsequently purified using a MinElute Gel Extraction Kit (Qiagen, Hilden, Germany) following the manufacturer’s instructions. After that, the amplicons were cloned using a CloneJET PCR Cloning Kit (Thermo Scientific, Waltham, MA, USA) and transformed into DH10B competent cells (Thermo Scientific, Waltham, MA, USA), according to the manufacturer’s instructions. The positive clones were screened by colony PCR using insert-specific primers (pJET1.2 forward and reverse sequencing primers, provided along with the kit). Colony PCR was performed according to the manufacturer’s instructions supplied along with the cloning kit. Following the screening of the positive clones, plasmid DNA was isolated using an Ultraclean^TM^ plasmid prep kit (Mo Bio, Jefferson City, USA), according to the manufacturer’s guidelines and sent for custom DNA sequencing (Eurofins Genomics, Ebersberg, Germany). The integrity of the plasmid DNA was measured by running the samples on a 1% agarose gel, prior to sending the samples for sequencing.

### 2.6. Three-Dimensional Structural Validation and Visualization 

SWISS-MODEL was used to predict the 3D structures of the wild type (WT) *Cyp51* gene (GenBank Acc. No. AMD11308.1) and mutant type (MT) *Cyp51* [14]. The best structure was selected based on the QMEAN scoring function. The structures were further validated and assessed in the PROCHECK server for the error value and the Ramachandran plot. All of the predicted 3D structures were visualized by Chimera 1.15rc.

### 2.7. Molecular Docking and Simulation Studies

The PyRx 0.8 Autodock Vina module (grid box size was 68.41 for x, 61.57 for y- and 64.51 for z-axis) was used to perform a series of protein-ligand docking studies to identify the most reliable binding pose and energy. Protein-ligand docking was visualized and analysed by Chimera 1.15cr at first, and then, more detailed analyses for interacting amino acid residues with the ligands were performed with BIOVIA Discovery Studio Visualizer. Molecular dynamics simulations were performed at 300 K with the GROMACS 2020.1 software package in the Ubuntu Linux system by using the OPLS-AA force field (protein only system) and CHARM36 (protein-ligand system) force field. All systems were packed in a 10 Å dimension cubic water box by using the gmx editconf module for boundary condition setup and solvation with the gmx solvate module. Further, the simulation system was immersed in a simulation box with a point charge SPC216 (protein only) and TIP3P (protein-ligand) water model. For neutralization of simulation system, Na^+^ and Cl^−^ ions were added to the system box, and the physiological system was also maintained (0.15 M) using the gmx genion module. For energy minimization, the steepest descent method was used. The maximum step size along a 0.01 nm gradient had a maximum of 50,000 steps. Furthermore, the simulation system was equilibrated at a constant temperature of 300 K, using the NVT and NPT ensemble simulation processes for 100 ps. Initially, the modified Berendsen thermostat with no pressure coupling was applied for the NVT (constant number of particles, volume, and temperature) canonical ensemble, and then, the Parinello–Rahman method pressure of 1 bar (P) was applied for the NPT ensemble (constant number of particles, pressure, temperature). The final simulations were performed for each system for 10 ns, where leap-frog integrator was applied for the trajectory time evolution [33,34].

### 2.8. MD Trajectories Analysis

All trajectories were analysed by using a trajectory analysis module integrated in the GROMACS 2020.01 simulation package, python3, matplotlib, qtgrace, VMD, and Chimera software. The trajectory files were first analysed by using GROMCAS tools, gmx rmsd, gmx rmsf, gmx gyrate, gmx S_ASA_, gmx, hbond, gmx energy for extracting the graph of root-mean square deviation (RMSD), root-mean square fluctuations (RMSFs), radius of gyration (Rg), solvent accessible surface area (S_ASA_), hydrogen bond, potential energy, kinetic energy, and enthalpy.

## 3. Results

### 3.1. Fungicide Sensitivity Assays

Overall, three different resistant strains (R2, R5, and R10, against propiconazole, prochloraz, and epoxiconazole, respectively) and two susceptible strains (S3 and S4) were identified and used for further analysis. The EC_50_ values of fifty strains are shown in Table S1. All three fungicides were found to control the growth of S3 and S4 strains, successfully. The EC_50_ value of R2 (14.27 μg/mL) was found to be 126 times greater than that of susceptible strains (average EC_50_ of S3 and S4 is 0.11 μg/mL), which indicates a high level resistance against propiconazole (Table 2). Similarly, high resistance factors were obtained for R5 (RF = 153) and R10 (RF = 119), indicating a high level of resistance against prochloraz and epoxiconazole, respectively (Table 2). The isolate/s with EC_50_ > 1.0 ug/mL were considered to be resistant. Based on this, we had screened the collected *C. beticola* isolates from the Czech Republic. Thereafter, based on the highest EC_50_ value and visual observation of mycelial growth reduction, we had opted to continue the study with the highest EC_50_ as resistant and EC_50_ ≤ 0.5 ug/mL values for all three DMI fungicide considered as sensitive isolates. Additionally, patterns of reduced sensitivity between the DMI fungicides were also observed in all the resistant strains. In summary, R2, R5, and R10 were considered resistant against propiconazole, prochloraz, and epoxiconazole.

### 3.2. CbCyp51 Gene Mutations 

**Figure 1:**
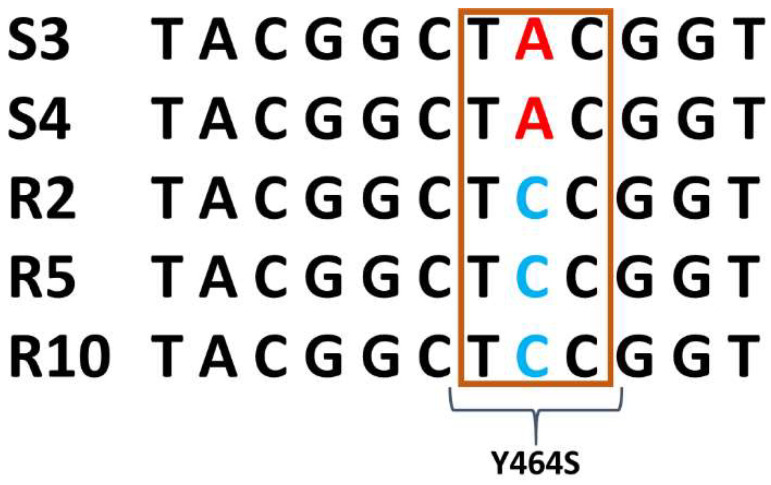
Multiple sequence alignment is multiple sequence alignment of the varied region of *Cyp51* gene of *Cercospora beticola* Sensitive (S3 & S4) and Resistant (R2, R5 & R10) strains. #S: sensitive biotypes; #R: resistant biotypes.

Based on our BLAST analysis, the obtained *Cyp51* gene (following the sequencing) corresponds to Cyp51A isoform (99.94% similarity). According to the calculated EC_50_ values against propiconazole, prochloraz, and epoxiconazole, all three strains (R2, R5, and R10, respectively) could be considered resistant and two sensitive strains. The first hypothetical resistance mechanism for shifting DMI and increased EC_50_ values, would be single nucleotide polymorphisms in the target of *Cyp51* gene. A relationship of the strain with the EC_50_ values were used to visualize this. The EC_50_ values of the strain carrying the wild type genotype has a significantly lower EC_50_ compared to the EC_50_ values of the strains with point mutation *Cyp51* gene. The primer pairs F3 and R3 and F4 and R4 resulted in 1453 bp and 1229 bp amplicons, respectively (gene accession number KU665583.1). The PCR products were evaluated in a 1.5% agarose gel, where a single band was obtained for each primer pair (data not shown). A point mutation in all three strains with a high level of resistance (R2, R5, and R10) was found. A single A to C exchange at nucleotide 1391 resulted in the exchange of tyrosine (Y) to serine (S) at codon position 464 (Y464S) of *Cyp51*. Amplification of the *C. beticola* 14α-demethylase gene with the F3 and R3 and F4 and R4 primers detected the Y464S mutation in all three resistant strains (Figure 1).

### 3.3. Impact of the Y464S Mutation on Fungicide Binding

A decrease in the binding affinity value and an increase in the S_ASA_ were predicted for all DMI fungicides, in the case of *Cyp51* MT-fungicide interaction, when compared with the *Cyp51* WT-fungicide interaction (Supplementary Figures S1–S3). This indicates a change in the conformation of the *Cyp51* protein, which might have occurred due to the Y464S mutation.

**Figure 2:**
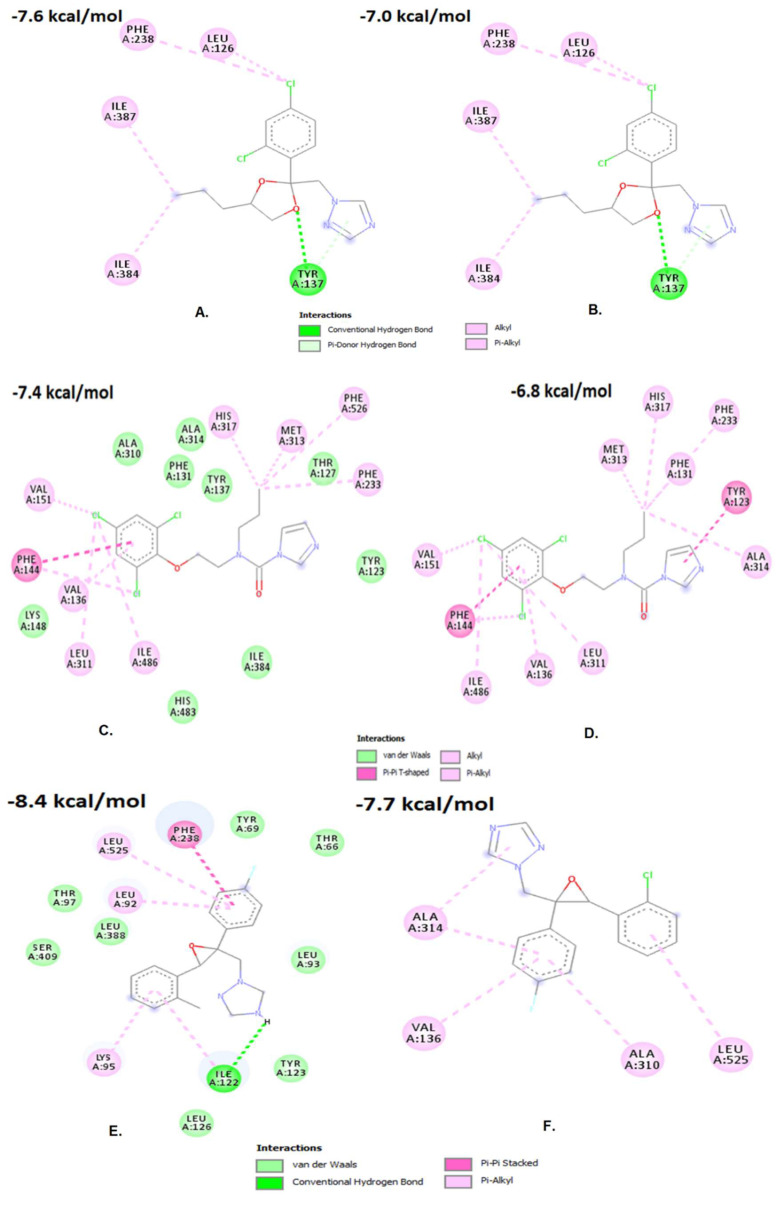
Results for interacting amino acid residues of *Cyp51* gene of *Cercospora beticola*. (**A**). *Cyp51*_WT and propiconazole binding, (**B**). *Cyp51*_MT and propiconazole binding; (**C**). *Cyp51*_WT and prochloraz binding, (**D**). *Cyp51*_MT and prochloraz binding; (**E**). *Cyp51*_WT and epoxiconazole binding, (**F**). *Cyp51*_MT and epoxiconazole binding. The values mentioned in the figures are binding affinity values. “WT” stands for wild type genotype and “MT” stands for mutant type genotype.

Overall, 18 amino acid residues were predicted to be directly involved in propiconazole binding in the *Cyp51* WT-fungicide interaction (Figure 2). However, for *Cyp51* MT-fungicide interaction, fewer amino acid residues (only eleven) were predicted to be directly involved in propiconazole binding (Figure 2). No amino acid residues were found to be involved in hydrogen bonding in either case (Figure 2). Moreover, our analysis detected seven interacting amino acid residues (Thr127, Tyr137, Lys148, Ala310, Ile384, His483, and Phe526) in the wild type but absent in the *Cyp51* MT (Figure 2). A total of five amino acid residues (Phe238, Leu126, Ile387, Ile384, and Tyr137) were predicted to be directly involved in prochloraz binding, irrespective of WT or MT (Figure 2). Overall, 13 amino acid residues were predicted to be directly involved in epoxiconazole binding for the wild *Cyp51* WT-fungicide interaction (Figure 2). Among these 13 amino acid residues, only Ile122A, was detected to be involved in hydrogen bonding (Figure 2). Although, in the case of the MT *Cyp51*-fungicide interaction, only four amino acid residues were predicted to be directly involved in epoxiconazole binding (Figure 2). Additionally, no amino acid residue was found to be involved in the MT *Cyp51*-fungicide interaction. In total, two types of short-range energies Lennard-Jones (LJ-SR), Coulomb (Coul-SR) and the sum of both during the simulation were also considered. In all cases, we predict the overall sum of the short-range energies in the WT-fungicide interaction is higher than that of the MT-fungicide interaction, thereby confirming the impact of the Y464S mutation on fungicide binding (Supplementary Table S2). 

### 3.4. Cyp51 Gene Expression Analysis

**Figure 3:**
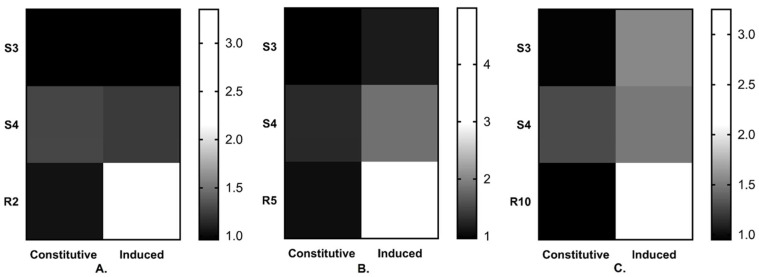
Analysis of gene expression variation of *Cyp51* gene of *Cercospora beticola*. The gene expression values are in terms of 2^−ΔΔCt^ values. Constitutive and fungicide-induced over-expression under (**A**). Propiconazole treatment, (**B**). Prochloraz treatment and (**C**). Epoxiconazole treatment. In this figure, fungicide-exposed samples are termed as “induced”. “S stands for sensitive biotype and R stands for resistant biotype”.

Another possible resistance mechanism associated with reduced sensitivity towards DMI could be constitutive or fungicide-induced over-expression of the *Cyp51* gene. To study this hypothesis, an experiment of Bolton et al. 2012 was performed. The expression level of *Cyp51* gene from treated and non-treated strains was determined by RT-qPCR. The constitutive and fungicide induced gene over-expression analysis indicated that the *Cyp51* gene expression was significantly induced after fungicide treatment for all three tested resistant strains (Figure 3, Supplementary Table S3).

## 4. Discussion

Cercospora leaf spot disease caused by *C. beticola* is the most predominant foliar leaf disease in sugar beet cultivation, worldwide [2,35]. The management tools used to control *C. beticola* infections are resistant varieties, cultural practices, and different groups of fungicides e.g., DMI, MBCs, and QoIs groups of fungicides, are efficiently used to manage CLS infections [36,37]. According to FRAC, DMIs have been categorized as medium risk fungicides for fungicide resistance development. Research and better understanding of the fungicide resistance mechanisms, mediating this sensitivity reduction will be helpful for a sustainable and appropriate disease and fungicide-resistance management. However, despite of the effectiveness of these fungicides, lack of effective pest management strategies along with overreliance on DMIs have led to the evolution of fungicide resistance in plant pathogenic fungi including *C. beticola* populations, globally [8,20,29,38,39]. 

Our study is the first detailed study from the Czech Republic (central Europe) to elucidate the mechanism of DMI fungicide resistance in *C. beticola* strains. In the study, 50 populations were examined for three DMI fungicide sensitivities, from which three strains with a Y464S mutation and two wild type strains were found. Based on sequence similarity results (as obtained from the BLAST analysis), the sequenced *Cyp51* reported in this article corresponds to *Cyp51*A. Hence, it can be concluded that *Cyp51A* gene is the main gene responsible for resistance in these biotypes. Our result is similar to obtained by other researchers [11]. Mutation Y464S was found in all three-resistance strains, but they should be considered in future monitoring and examination since they also occur in conserved regions of *Cyp51* gene [8]. Mutations within sterol P450 14α-demethylase (*Cyp51*), a DMI target enzyme, are known to associate with reduced sensitivity to DMI fungicides in *C. beticola* [40] and some other phytopathological fungi e.g., *Rhynchosporium secalis* [41]. Moreover, mutation Y464S is equivalent to the single nucleotide polymorphisms Y461S in the closely related fungus *Z. tritici*. This mutation was reported in different DMI modified *Z. tritici* strain but additionally, this point mutation seems to be also an interesting site for some other amino acid exchanges like Y461H [14,42]. As shown for *Z. tritici*, different single nucleotide polymorphism was associated with varying levels of sensitivity losses towards different DMI [18,43,44].

Structural study of the protein revealed that the mutations occur either inside evolutionary or near conserved regions. The point mutation Y464S occurs 10 amino acids upstream of the region FXXGXXXCXG, a highly conserved heme binding domain [8]. Similarly, we also detected the Y464S mutation in all resistant strains. The mutation Y464S had been reported in several strains of *C. beticola* which were associated with resistance against DMI fungicides [8]. This led to hypothesis that the Y464S mutation in *Cyp51* gene might associate with resistance in MT strains, and hence WT strains without mutation are sensitive to the DMI group of fungicides. Although there are some earlier reports on the association between target gene overexpression and reduced fungicide sensitivity [19,45,46], however, the first evidence of such mechanisms in *C. beticola* has been reported by Nikou. D. et al., in 2009 [39] and later by Bolton in 2012 [29]. They found a constitutive over-expression of *Cyp51* gene of resistant *C. beticola* strains to be responsible for reduced sensitivity and increased EC_50_ values [23,29]. Hence, fungicide-induced over-expression of the *Cyp51* gene was also studied, to investigate whether this mechanism is also involved in fungicide resistance [47]. Over-expression of the P450-dependent sterol 14α-demethylase-encoding gene has been recognized as a key strategy of DMI fungicide-resistance [15]. In this case, we found no significant effect of the DMI on the *Cyp51* gene expression for susceptible strains. However, for resistant strains, we found a significant change in the gene expression prior and after fungicide application. In the R2 strain, we detected 3.21-fold overexpression, after application of propiconazole. The R5 and R10 strains showed ~4.7 and ~3.4-fold overexpression, respectively, after fungicide treatment. Hence, we conclude that the Y464S point mutation along with fungicide-induced over-expression of the *Cyp51* gene confer resistance against DMI fungicides in *C. beticola*. 

Theoretically, the efficiency of a drug molecule will be the best when its biologically active conformation fits with that of its target’s binding pocket [48]. With the change in the conformation of the binding pocket, the drug may become less effective, thus developing resistance [49]. Molecular docking with *Cyp51* gene structure models was used to understand difference between MT and WT biotypes for DMI fungicides in *C. beticola* [50]. We demonstrated molecular docking models along with the binding affinity values [51]. The binding affinity values of the *Cyp51* genotype from the resistant strains were decreased when compared with that of the sensitive strains [52]. Molecular docking accompanied by simulation studies showed that in all cases, the Y464S mutation influences the conformation of the *Cyp51* genotype, which is one of the molecular mechanisms for development of resistance to DMI fungicides [52]. Overall, 18 amino acid residues were predicted to be directly involved in propiconazole binding in the *Cyp51* WT-fungicide interaction. However, for *Cyp51* MT-fungicide interaction, fewer amino acid residues (only eleven) were predicted to be directly involved in propiconazole binding. No amino acid residues were found to be involved in hydrogen bonding in either case. Moreover, our analysis detected seven interacting amino acid residues (Thr127, Tyr137, Lys148, Ala310, Ile384, His483, and Phe526) involved in the wild type but absent in the *Cyp51* MT. These amino acid residues might be important for propiconazole binding. In total, five amino acid residues (Phe238, Leu126, Ile387, Ile384, and Tyr137) were predicted to be directly involved in prochloraz binding. These amino acid residues might be important for prochloraz binding. Overall, 13 amino acid residues were predicted to be directly involved in epoxiconazole binding in the case of the *Cyp51* WT-fungicide interaction. Among these 13 amino acid residues, only Ile122A was detected to be involved in hydrogen bonding. However, in the case of the MT *Cyp51* fungicide interaction, only four amino acid residues were predicted to be directly involved in epoxiconazole binding. Additionally, no amino acid residue was found to be involved in the MT *Cyp51* fungicide interaction. Thus, we can predict that hydrogen bonds might have an essential role in fungicide binding. Hydrogen bonds are known to provide most of the directional interactions that involves molecular recognition [53]. Furthermore, two types of short-range energies Lennard-Jones (LJ-SR), Coulomb (Coul-SR) and the sum of both during the simulation were also considered. In all the cases, we predict the overall sum of the short-range energies in the WT-fungicide interaction is higher than that of the MT-fungicide interaction, thereby confirming the impact of the Y464S mutation on fungicide binding. Although, these hypotheses require further validation. Molecular docking studies with the homology-modelled proteins of interest and the existing fungicides can serve as an important prototype study. Earlier a structural rationale study was conducted for imidazole and triazole resistance associated with *Cyp51* mutations. They had modelled the wild-type and mutated variants of *Cyp51* from *Mycosphaerella graminicola.* They had predicted the probable binding orientations successfully [54]. Another study had also conducted molecular docking studies with *Cyp51* from *Colletotrichum truncatum* and DMI fungicides. They had assessed the effects of four amino acid variations (L208Y, H238R, S302A, and I366L) on *Cyp51*A- DMI fungicide binding. Their results showed that four alterations might reduce azole affinity [55]. However, these hypotheses require further validation. Not many studies with in-silico techniques have been conducted in the fungicide-resistance area of research. Hence, our current study will serve as an important prototype study. The previous studies along with ours will provide potential for an in silico screening system and reliable predictive approach to assess the probability of particular variants exhibiting resistance to particular azole fungicides. These studies will open up many possible opportunities leading to the discovery of new compounds with fungicidal properties.

## 5. Conclusions

We identified a point mutation in all three-resistant *C. beticola* strains correlating with reduced sensitivity towards DMI fungicides. This mutation was found in highly conserved domains of the target *Cyp51* gene, not only in *C. beticola* but also in other plant pathogenic fungi displaying reduced DMI sensitivity.

We found a stronger fungicide-induced over-expression of the *Cyp51* in strains with high EC_50_ values compared to low EC_50_ values strains.

The present study concludes that the population from the Czech Republic are developing resistance against the DMI group of fungicides. The findings from this study provide a basis for future research based on reverse genetics to elucidate the role of the target site mutations on the expression level of *Cyp51* and the DMI in *C. beticola*.

## Figures and Tables

**Table 1:** List of primers used in this study. Primer pairs F1-R1 and F2-R2 were used for copy number variation as well as gene expression analysis. Primer pairs F3-R3 and F4-R4 were used to isolate and clone partial *Cyp51* gene from *Cercospora beticola*.

Name	Sequence (5’ to 3’)	Amplicon Length (bp)	Annealing Temperature (°C)
F1	TCGTCTTCCACTTCGTACCC	172	58
R1	CCGTTCAGGATGAAGTCGTT		
F2	ACGGAGTTACCCACGTTGTC	174	58
R2	TCTCCTTGATGTCACGAACG		
F3	TCGTCTTCCACTTCGTACCC	1453	60
R3	CTCTCCCACTTCACAACAGC		
F4	GTGTTTGGCAAGGACGTCG	1229	61
R4	CTCTCCCACTTCACAACAGC		

**Table 2:** Results of fungicide sensitivity assays. EC_50_ values (in μg/mL) were calculated using Graph Pad Prism software (9.0.0), based on mean colony diameter and radial growth of each strain.

Strain	Propiconazole	Prochloraz	Epoxiconazole
S3	0.17 (±0.02)	0.19 (±0.02)	0.05 (±0.01)
S4	0.05 (±0.01)	0.32 (±0.22)	0.45 (±0.32)
R2	14.27 (±2.94)	2.29 (±0.23)	2.19 (±0.17)
R5	5.43 (±0.54)	44.01 (±5.85)	2.78 (±0.13)
R10	3.11 (±0.14)	4.36 (±0.42)	39.53 (±6.23)

## Data Availability

The data that support the findings of this study are available from the corresponding author upon reasonable request.

## Supplementary Materials

The following are available online at https://www.mdpi.com/article/10.3390/jof7121062/s1, Supplementary Table S1. Fungicide sensitivity of *Cercospora beticola* against three DMIs fungicides (propiconazole, prochloraz, and epoxiconazole). The EC_50_ values are in (μg/mL); Supplementary Figure S1. Solvent accessible surface area analysis during propiconazole-*Cyp51* interaction; Supplementary Figure S2. Solvent accessible surface area analysis during prochloraz-*Cyp51* interaction; Supplementary Figure S3. Solvent accessible surface area analysis during epoxiconazole *Cyp51* interaction; Supplementary Table S2. Lennard-Jones energies short-range (LJ-SR) and Coulombic potential within R-coulomb Coul-SR) between protein and fungicide molecule, during MD simulations; Supplementary Table S3. Analysis of gene expression variation. The relative gene expression values are in terms of 2^-ΔΔCt^ values. “*” denotes significant at 5% significant level.
