# Supplementary material for: Characterization of the Molecular Mechanisms of Resistance against DMI Fungicides in Cercospora beticola Populations from the Czech Republic
Source: J Fungi (Basel). 2021 Dec 11;7(12):1062. doi: 10.3390/jof7121062 (PMC8706352; doi:10.3390/jof7121062)
Supplement: Supplementary file 1 [file jof-07-01062-s001.zip › jof-1452848-supplementary.pdf]

Type of the Paper (Article)

Characterization of the molecular mechanisms of resistance against DMI fungicides in *Cercospora beticola* populations from the Czech Republic

Ram Kumar[1], Jana Mazakova[1], Asad Ali[1], Vishma Pratap Sur[2], Madhab Kumar Sen[3], Melvin D. Bolton[4], Marie Manasova[1], Pavel Rysanek[1], Miloslav Zouhar[1*]

[1]Department of Plant Protection, Faculty of Agrobiology, Food and Natural Resources, Czech University of Life Sciences Prague, Kamycka 129, 165 00, Prague 6, Suchdol, Czech Republic ; kumar@af.czu.cz (R.K.); mazakova@af.czu.cz (J.M.); aliasad@af.czu.cz (A.A.); manasova@af.czu.cz (M.M.); rysanek@af.czu.cz (P.R.)

[2]Laboratory of Reproductive Biology, Institute of Biotechnology of the Czech Academy of Sciences, BIOCEV, Prumyslova 595, 252 50 Vestec, Czech Republic; VishmaPratap.Sur@ibt.cas.cz

[3]Department of Agroecology and Crop Production, Faculty of Agrobiology, Food, and Natural Resources, Czech University of Life SciencesPrague, Kamycka 129, 165 00 Prague 6, Suchdol, Czech Republic;senm@af.czu.cz

[4]Northern Crop Science Laboratory, United States Department of Agriculture, 1307 18th St N, Fargo, ND, 58102, USA ; melvin.bolton@usda.gov

***Correspondence to:** zouhar@af.czu.cz

Supplementary Table S1. Fungicide sensitivity of *Cercospora beticola* against three DMIs fungicides (propiconazole, prochloraz, and epoxiconazole). The EC_{50} values are in (µg/mL).

**Isolates**	**Propiconazole**		**Prochloraz**		**Epoxiconazole**	
	**Average**	**Standard deviation**	**Average**	**Standard deviation**	**Average**	**Standard deviation**
7B_1 (S3)	0.17	0.02	0.19	0.02	0.05	0.01
1_6 (S4)	0.05	0.01	0.32	0.22	0.45	0.32
ST_8 (R2)	14.27	2.94	2.29	0.23	2.19	0.17
36_2 (R5)	5.43	0.54	44.01	5.85	2.78	0.13
M61_5 (R10)	3.11	0.14	4.36	0.42	39.53	6.23
7A_2	1.36	1.05	1.98	1.21	2.06	0.64
7A_3	1.22	0.04	2.06	0.56	1.14	0.93
7A_4	1.81	1.52	1.53	1.09	1.52	0.51
7A_5	1.29	0.59	ND	ND	1.61	0.35
3_2	0.90	0.54	1.96	0.65	5.72	8.22
3_3	1.24	1.00	1.17	0.25	1.06	0.70
3_4	2.71	1.54	1.10	0.80	ND	ND
3_5	0.62	0.54	0.47	0.37	1.18	0.34
4_2	1.84	0.92	1.48	0.22	ND	ND
4_3	1.13	0.19	1.53	1.24	0.84	0.40
4_4	0.24	0.08	0.61	0.53	1.29	0.35
5_2	1.22	0.62	2.05	0.94	1.49	1.19
5_3	1.47	0.66	1.48	0.38	1.06	0.51
5_4	0.95	0.68	0.98	0.90	1.47	0.20
5_5	0.35	0.25	0.75	0.34	1.22	0.50
vs_1	1.04	0.15	0.87	0.22	7.60	11.16
vs_2	0.97	0.27	ND	ND	3.99	3.46
vs_3	ND	ND	1.17	0.51	4.92	5.94
8_1	ND	ND	2.39	1.29	2.37	1.06
8_2	1.06	0.37	2.68	1.51	1.24	0.37
8_3	1.04	0.12	1.45	0.96	1.20	0.79
9_1	2.03	0.90	0.98	0.57	ND	ND
9_2	1.32	0.27	1.15	1.01	1.11	0.79
9_3	1.12	0.82	1.14	0.95	1.15	0.59
11_1	1.29	0.39	0.92	0.70	1.06	0.41
11_2	ND	ND	2.80	1.44	1.43	0.73
11_3	1.30	0.40	1.50	1.32	1.88	0.15

17_1	1.46	0.91	1.42	1.03	1.41	1.13
17_2	1.25	0.54	1.30	0.98	1.50	0.71
17_3	1.49	0.70	0.31	0.29	1.72	1.26
20_1	ND	ND	0.97	0.46	1.19	0.30
23_1	1.12	0.69	0.50	0.57	1.42	0.91
24_1	1.22	0.68	1.02	0.66	0.54	0.45
25_1	2.54	0.95	0.77	0.78	1.23	0.85
70_5	1.21	0.50	0.53	0.44	1.41	0.57
70_1	1.86	0.23	0.57	0.41	1.47	0.95
70_2	0.71	0.46	ND	ND	1.12	0.42
72_1	1.29	1.07	1.79	1.26	1.30	0.42
73_1	0.91	0.83	0.62	0.49	0.93	0.73
E_1	1.91	0.77	1.47	1.98	0.50	0.38
E_2	1.01	0.34	0.68	0.45	0.95	0.47
E_3	1.02	0.26	0.80	0.91	0.57	0.46
E_4	1.01	0.26	1.75	0.52	0.96	0.83
E_5	0.89	0.92	1.35	0.49	0.91	0.64
M61_1	0.97	0.56	0.83	0.95	1.13	0.72
M61_2	0.88	0.49	1.48	0.43	ND	ND

#ND: “not done”.
#S: sensitive biotype
#R: resistant biotype

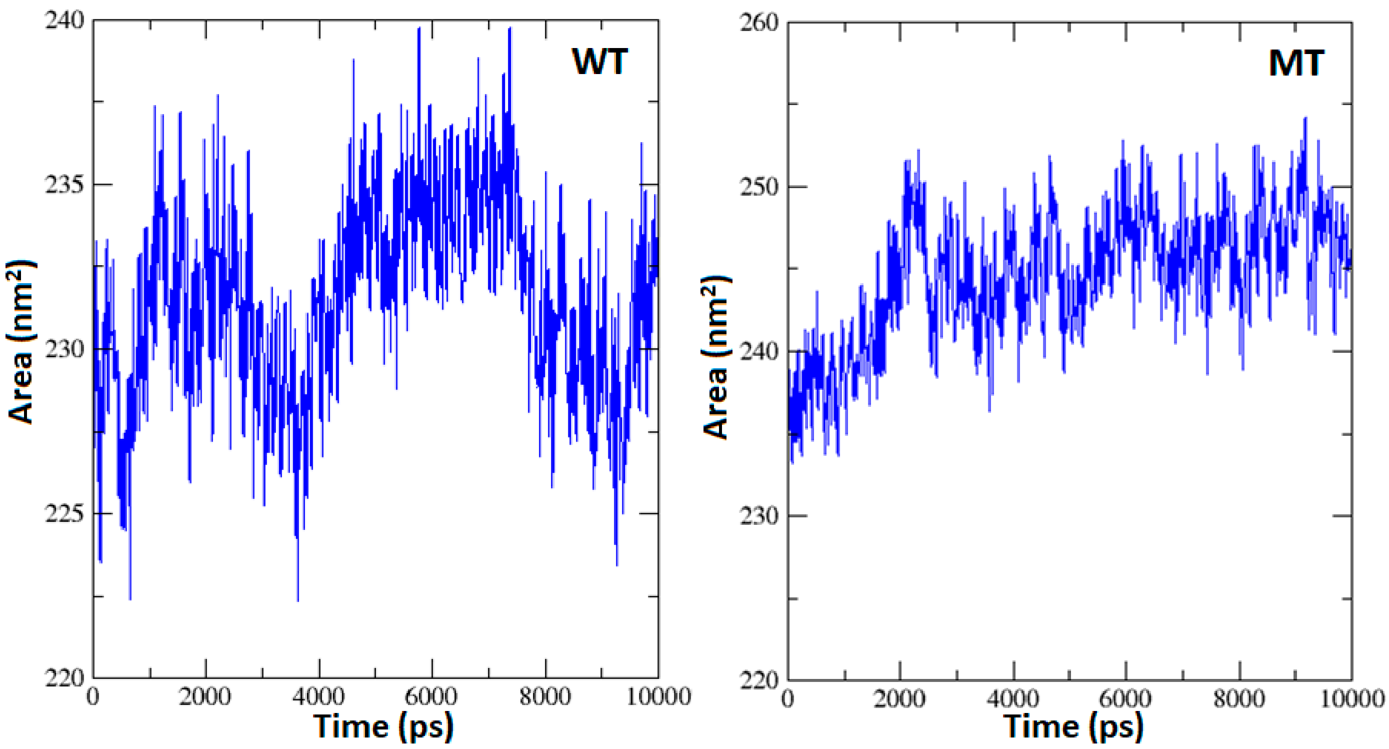

Supplementary Figure S1. Solvent accessible surface area analysis during propiconazole-*Cyp51* interaction between wild type (WT) genotype and mutant type (MT) genotype of *Cercospora beticola*.

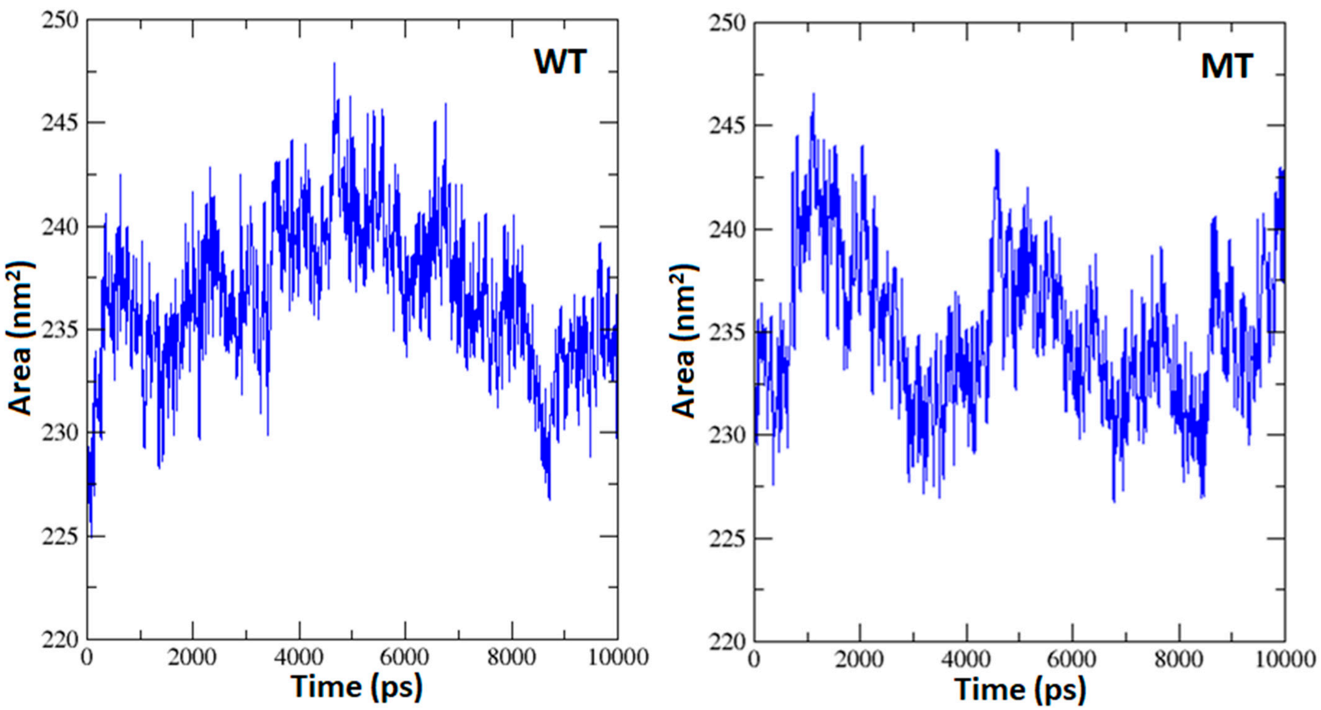

Supplementary Figure S2. Solvent accessible surface area analysis during prochloraz-*Cyp51* interaction between wild type (WT) genotype and mutant type (MT) genotype of *Cercospora beticola*.

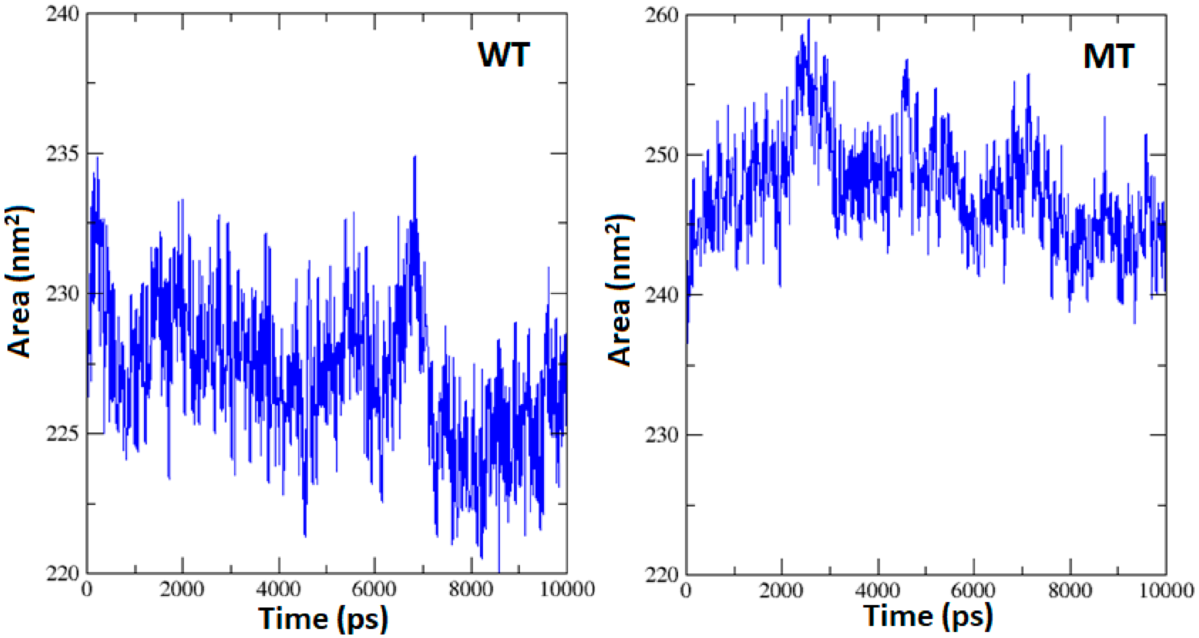

Supplementary Figure S3. Solvent accessible surface area analysis during epoxiconazole *Cyp51* interaction between wild type (WT) genotype and mutant type (MT) genotype of *Cercospora beticola*.

Supplementary Table S2. Lennard-Jones energies short-range (LJ-SR) and Coulombic potential within R-coulomb (Coul-SR) between *Cyp51* protein of *Cercospora beticola* and fungicide molecule, during MD simulations.

**Propiconazole**				
	**Wild Type**		**Mutant Type**	
**Energy type**	**Interaction energy (kJ/mol)**	**Estimated error**	**Interaction energy (kJ/mol)**	**Estimated error**
Coul-SR	-18.392	1.6	-23.0891	2.7
LJ-SR	-162.367	2.4	-144.432	2.2
**Prochloraz**				
	**Wild Type**		**Mutant Type**	
**Energy type**	**Interaction energy (kJ/mol)**	**Estimated error**	**Interaction energy (kJ/mol)**	**Estimated error**
Coul-SR	-18.5526	1.5	-13.4189	3.2
LJ-SR	-140.738	1	-121.644	1.8
**Epoxiconazole**				
	**Wild Type**		**Mutant Type**	
**Energy type**	**Interaction energy (kJ/mol)**	**Estimated error**	**Interaction energy (kJ/mol)**	**Estimated error**
Coul-SR	-16.2723	3.7	-7.09995	1
LJ-SR	-123.091	3.1	-119.641	1.3

Supplementary Table S3. Analysis of gene expression variation of *Cyp51* gene of *Cercospora beticola*. The relative gene expression values are in terms of $2^{-\Delta\Delta Ct}$ values. "*" denotes significant at 5% significant level.

Isolates	Propiconazole			
	**Constitutive expression**	**Fungicide-induced expression**	**Significant at 5% significance level**	**Fold change**
S3	0.96 (± 0.03)	0.96 (± 0.34)	NS	NA
S4	1.28 (± 0.25)	1.23 (± 0.11)	NS	NA
R2	1.04 (± 0.11)	3.35(± 0.38)	*	3.21
**Isolates**	**Prochloraz**			
	**Constitutive expression**	**Fungicide-induced expression**	**Significant at 5% significance level**	**Fold change**
S3	0.96 (± 0.03)	1.17 (± 0.32)	NS	NA
S4	1.28 (± 0.25)	1.84 (± 0.36)	NS	NA
R5	1.07 (± 0.17)	4.98 (± 0.64)	*	4.67
**Isolates**	**Epoxiconazole**			
	**Constitutive expression**	**Fungicide-induced expression**	**Significant at 5% significance level**	**Fold change**
S3	0.96 (± 0.03)	1.56 (± 0.56)	NS	NA
S4	1.28 (± 0.25)	1.49 (± 0.08)	NS	NA
R10	0.95 (± 0.09)	3.25 (± 0.67)	*	3.43

#NS: "non-significant"

#NA: "not applicable"

#S: sensitive biotype

#R: resistant biotype
